# Impact of drug-resistant tuberculosis treatment on hearing function in South African adults: Bedaquiline versus kanamycin

**DOI:** 10.4102/sajcd.v68i1.784

**Published:** 2021-01-26

**Authors:** Katijah Khoza-Shangase, Marina Prodromos

**Affiliations:** 1 Department of Speech Pathology and Audiology, Faculty of Humanities, University of the Witwatersrand, Johannesburg, South Africa

**Keywords:** bedaquiline, drug-resistant tuberculosis, hearing, kanamycin, monitoring, ototoxicity

## Abstract

**Background:**

Ototoxicity linked to medications used to treat tuberculosis (TB) remains a global challenge.

**Objectives:**

The aim was to describe the audiological function in a group of adults with drug-resistant tuberculosis (DR-TB) on bedaquiline (G-BDQ) treatment attending a TB hospital in South Africa and compare this with patients on kanamycin (G-KCIN).

**Methods:**

A quantitative paradigm was adopted within a non-experimental retrospective record review design. The sample consisted of 30 records of adults with DR-TB between the ages of 18 and 50 years, recruited from a Tropical Diseases Hospital in South Africa. Data were analysed through both descriptive and inferential statistical measures.

**Results:**

Clear and statistically significant differences in the audiological function were found between the two groups. The group receiving G-KCIN presented with ototoxicity that was clearly demonstrated by sensorineural hearing loss of high-frequency worsening of thresholds in over 73% of the records, which was statistically (*p* < 0.05) and clinically significant, over the three testing sessions, demonstrating the cumulative effects of dosage. Increased evidence of tinnitus was also found in this group. The group receiving G-BDQ presented with neither statistically (*p* > 0.05) nor clinically significant changes in hearing thresholds across all frequencies over the same monitoring timeframe. Additionally, only one report (7%) of tinnitus was found in this group.

**Conclusion:**

The results indicating that bedaquiline does not cause hearing loss when compared with G-KCIN highlight the need for increased availability of bedaquiline for the treatment of DR-TB within the South African context, to preserve both the quantity and quality of life of those infected.

## Introduction

The World Health Organization (WHO, 2020) reports that multidrug-resistant tuberculosis (DR-TB) remains a public health crisis and a health security threat globally, with estimates of 484 000 new cases with resistance to rifampicin, which is the most effective first-line drug. South Africa reported an estimated incidence of 301 000 cases of active TB in 2018, with 11 000 people having become ill with MDR or rifampicin-resistant TB in 2018 (WHO, 2019a). Factors such as human immunodeficiency virus (HIV), insufficient infection control in hospitals and clinics and a public health infrastructure not equipped to ensure that patients complete the TB treatment courses have contributed to the rise in the incidence of TB in South Africa (Murphy, 2008; WHO, 2019a, 2020). As with the HIV 90-90-90 targets, South Africa committed to the Global Plan to End TB 2016–2020 strategy, which also involves implementing the 90-90-90 strategy. This entails that 90% of all TB cases should be found and given effective treatment, that 90% of the TB cases in key populations should be found and provided with appropriate treatment and that there should be a 90% treatment success rate amongst people identified as needing treatment (WHO, 2020). Khoza-Shangase (2020a) suggested that there should be a fourth 90%, which includes maintenance of at least 90% of quality of life, where hearing preservation can be located as it has an impact on quality of life.

Traditional DR-TB medications have known audiological and non-audiological effects (Khoza-Shangase, Lecheko, & Ntlhakana, 2020). Aminoglycosides, such as kanamycin, amikacin and capreomycin, that are often used to treat DR-TB lead to ototoxicity (Khoza-Shangase & Stirk, 2016; Petersen & Rogers, 2015; Ramma, Schellack, & Heinze, 2019). Ototoxicity can be defined as the tendency of certain therapeutic agents and other chemical substances to cause functional impairment through cellular degeneration of the inner ear hair cells and neurons of the vestibulocochlear nerve (Bisht & Bist, 2011, p. 255). Drugs that have been found to induce ototoxicity include aminoglycosides, cisplatin, loop diuretics, quinine, non-steroidal anti-inflammatory drugs and anti-retroviral therapy (Khoza-Shangase, 2011, 2014; Paken, Govender, Pillay, & Sewram, 2016; Ramma et al., 2019).

The ototoxicity effects are first seen in the damage of the outer hair cells at the basal end of the cochlea and then progress towards the apical cochlear region. This leads to a progressive high-frequency hearing loss, and then a subsequent progressive loss towards lower-frequency hearing loss as well (Appana, Joseph, & Paken, 2016). These effects can be accompanied by other symptoms, such as tinnitus and balance disturbances (Hollander, Joubert, & Schellak, 2020; Khoza-Shangase, 2020a). Tinnitus can also be a side effect of ototoxic drugs as the drug has the potential to cause toxic reactions to the inner ear structures, such as the cochlea, vestibule, semi-circular canals and otoliths (Mudd & Glatz, 2018).

Drugs such as kanamycin (G-KCIN), a second-line DR-TB injectable, used to treat patients with DR-TB have been shown to suppress cochlear activity, causing ototoxicity in the form of permanent, bilateral, mild-to-moderate and sensorineural hearing loss (Gamit, 2014; Ramma et al., 2019). Ototoxic hearing loss is typically bilateral and symmetrical; however, rare cases of asymmetrical hearing loss have been reported (Rachana & Shabnam, 2017). Such hearing loss negatively impacts individual’s communication abilities and their quality of life (Cone et al., 2018). This is the motivation for highlighting the value of preventive ear and hearing care, where ototoxicity monitoring is performed at an early stage to identify any ultra-high-frequency changes to allow for preventive intervention prior to permanent damage in the speech frequencies (Appana et al., 2016; Khoza-Shangase & Stirk, 2016; Khoza-Shangase et al., 2020).

A number of factors influence the individual’s susceptibility to ototoxicity. These include dosage, method of drug administration, duration of treatment, renal function, age and so on (Bisht & Bist, 2011). Modongo et al. (2014) found that as dosage and duration of aminoglycoside treatment increased, so did the risk of ototoxicity. With the known negative side effects of current treatment options for TB treatment on hearing function, exploration of possible effects of newer treatment options is essential. This forms part of benefit–risk evaluations, which Khoza-Shangase (2017) argues is important for low- and middle-income countries (LMICs) to continually engage in. Within the South African context, the introduction of the new antibiotic, bedaquiline (BDQ), as one of the treatment options has raised implications for the audiologist as limited evidence on its possible effects on auditory function exists. This is particularly important as significant toxicity of second-line drugs in this field has been the norm (Lienhardt & González-Angulo, 2016; WHO, 2019b).

Bedaquiline is a diarylquinoline that has been reported to act by specifically inhibiting mycobacterial adenosine triphosphate synthase and has a long half-life of approximately 5 months (Brigden, Hewison, & Varaine, 2015). This drug, a 100-mg tablet for oral intake, is the first new anti-TB drug to be approved since 1998 and the first to be introduced, especially for the treatment of DR-TB in combination with other drugs (Deoghare, 2013). South Africa initially rolled out BDQ in small amounts across the country. Subsequently, in June 2018, the South African Department of Health announced the official rollout of BDQ on a much larger scale as a replacement for current aminoglycoside treatments for adolescents and adults.

The most common side effects that have been reported with BDQ treatment include nausea, arthralgia, headaches, haemoptysis, chest pain, anorexia and rash (Deoghare, 2013). Additionally, BDQ has also been shown to cause toxicity of the liver in some cases (Deoghare, 2013), and hence there is a need for investigating hearing function in patients taking the medication. Lienhardt and González-Angulo (2016) confirmed that safety concerns of BDQ treatment for DR-TB included hepatotoxicity and excess mortality. These authors, however, stress that despite the safety concerns, the benefits of BDQ treatment for DR-TB patients outweigh the risk and harm.

As far as hearing function is concerned, Khoza-Shangase (2017) argues that despite the curative benefits that one may gain from the potentially ototoxic medication, the hearing loss that may occur as a result may be a persistent or significant disability to the person affected, minimising the life-sustaining benefits of the drug for the individual affected. Thus, with appropriate ototoxicity monitoring, the patient’s hearing loss can be minimised and/or its impact mitigated as much as possible (Khoza-Shangase et al., 2020; Petersen & Rogers, 2015).

Early identification of ototoxicity allows for preventive treatment modifications to be instituted, thus preventing further hearing loss (Khoza-Shangase et al., 2020). Additionally, ototoxicity monitoring provides an opportunity for counselling and management during and post-treatment (Khoza-Shangase & Masondo, 2020), which has benefits for the medical and audiological management in terms of treatment adherence (Khoza-Shangase, 2017). Management may include counselling the patients and their family, teaching them appropriate communication strategies, as well as providing audiological rehabilitation and amplification, such as hearing aids and cochlear implants (Petersen & Rogers, 2015).

The current study, therefore, aimed to describe the audiological functioning in adults with DR-TB on G-KCIN treatment and compared that with those on BDQ treatment. In view of the increasing prevalence of DR-TB and the need for evidence-based benefit or risk evaluations in resource-constrained contexts like South Africa, investigating potential risks of new medications remains a priority.

## Methods and materials

### Aim

The primary aim of this study was to describe the audiological function in a group of adults with DR-TB on bedaquiline treatment (G-BDQ), who are attending a TB hospital in South Africa, and compare this with patients on G-KCIN treatment. Specific objectives included describing and comparing the hearing function of both groups at baseline, 1–2 months and 3–6 months post-commencement of treatment, whilst establishing the occurrence of tinnitus in both groups after commencement of treatment.

## Research design

A quantitative paradigm was adopted, and the research was conducted within a non-experimental retrospective record review design (Vassar & Holzmann, 2013). As a result of the fact that bedaquiline was being carefully introduced at the time of data collection, only a small sample size of participants who had completed the treatment could be collected. The sample, therefore, consisted of 30 medical and audiological records of adults with DR-TB between the ages of 18 and 50 years with a mean age of 35.2 years. The age limits of 18–50 years were chosen to control for the effects of developing cochlear function on ototoxicity, as well as presbycusis (Arvin, Prepageran, & Raman, 2011; Khoza-Shangase, 2020a). The sample was recruited from a Tropical Diseases Hospital in South Africa, and was equally distributed and age-matched (15 participant records for the G-KCIN and 15 for the G-BDQ). This is a specialised DR-TB treatment facility in Johannesburg, which also has an established research unit. The records were selected by using a convenience sampling technique with specific selection criteria. The following selection criteria were utilised:

### Inclusion criteria

Selected files had to:

- be from 2014 to 2018;
- be of patients aged 18–50 years;
- have a confirmed positive TB status, specifying the type of TB and respective treatment; and
- be of patients on BDQ treatment or on KCIN treatment.

Participants presenting with acquired audiological problems because of other causes during the data collection period were excluded from the study. This included causes such as trauma and infections including otitis media, meningitis, to name a few.

The medical record reviews were conducted by using a self-developed data-capturing sheet, which included age, gender, otoscopic findings, tympanogram type, tinnitus reports, air conduction threshold results and the medications the patients were on. This information was collected at all three monitoring sessions.

### Data analysis

Data were analysed through both descriptive and inferential statistical measures, and a comparative analysis of both groups was conducted. After capturing of all data on an excel spreadsheet, descriptive statistics was used to describe the basic features of the data, allowing the researchers to provide basic summaries about the sample (Trochim, 2002). The data were then organised graphically, in the form of tables, and line graphs. For inferential statistics, the 95% confidence level was used throughout. Inferential statistics in the form of analysis of variance (ANOVA) was used to establish statistical significance in changes of hearing thresholds over the specified periods of treatment with BDQ and KCIN (Trochim, 2002). Then, a two-sample, one-tailed *t*-test was carried out to identify the difference in hearing status between the two groups.

For the findings to be considered as having resulted in a significant ototoxic shift, pure-tone air conduction had to meet one of the three criteria, as specified by the American Academy of Audiology Position Statement and Clinical Practice Guidelines (2009):

- 20 dB decrease at any one test frequency;
- 10 dB decrease at any two adjacent test frequencies; and
- no response at three consecutive frequencies.

All analyses were conducted over the three sessions where data from baseline were compared with those in two subsequent sessions (inter-comparison) and then between the two groups (intra-comparison).

### Reliability and validity

To minimise participant variables from impacting on the reliability and validity of results, any records indicating the patient as being ‘unreliable’ and/or denoting ‘questionable reliability’ were excluded (Gautschi-Mills, Khoza-Shangase, & Pillay, 2019). The researcher also worked on the assumption that the qualified audiologists who ran the clinic adhered to minimum standards, were able to identify and report any unreliable responses and that they adhered to best practice in terms of test protocols, sound proofing and equipment calibration during the time when the records reviewed were completed (Khoza-Shangase, 2020a).

### Ethical consideration

Prior to the study being conducted, ethical clearance was obtained from the Human Research Ethics Committee of the University (Protocol Number: M180271). It was the researchers’ duty to ensure that basic ethical guidelines were adhered to as set out by the South African Medical Research Council Ethics Committee (2010), and furthermore the work adhered to the Helsinki Declaration of 1975, as revised in 2013.

## Results

### Description of the sample

The study comprised 30 age- and gender-matched participants between the ages of 18 and 50 years, with a mean age of 35.2 years. All of the participants had been diagnosed with DR-TB, with one half on G-KCIN injectable treatment and the other half on G-BDQ treatment.

### Ototoxicity monitoring in the kanamycin treatment

**FIGURE 1:**
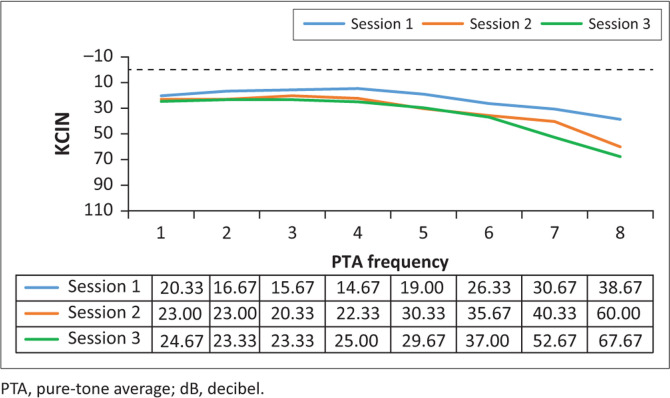
Mean left pure-tone audiometry results (in decibel) for the kanamycin treatment, at the three different sessions (*n* = 15).

**FIGURE 2:**
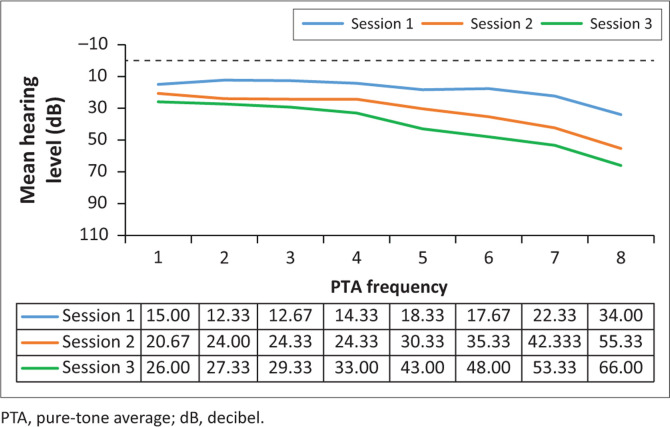
Mean right pure-tone audiometry results (in decibel) for the kanamycin treatment, at the three different sessions (*n* = 15).

Analysis of the results for this group (*n* = 15) involved comparing the pure-tone threshold results at all three sessions to determine whether any changes occurred during the period when they were receiving G-KCIN treatment. The pure-tone audiometry results in terms of means are depicted in Figures 1 and 2.

Close inspection of the results (Figures 1 and 2) for pure-tone audiometry revealed mean threshold shifts between all treatment sessions, gradually becoming worse, particularly at higher frequencies, therefore, indicating that there had been a steady decline in hearing function, which occurred over time. The right ear results appeared to be slightly worse than the left ear results, with thresholds shifting from an average of 34–66 dB at 8 kHz in that ear over the treatment period. Close inspection of the results indicated clinically significant shifts at high frequencies bilaterally right from session 2, with 73.33% of the patients exhibiting a shift from baseline to their first follow-up in the left ear, whilst 80% had a significant shift in the right ear at this session. The thresholds worsened with progressing time on treatment.

Results of statistical analysis of pure-tone audiometry by using ANOVA are depicted in Table 1.

**TABLE 1:** Results of analysis of variance for pure-tone audiometry for the kanamycin treatment, indicating level of significance.

Frequency	*p*	F	Α	Frequency	*p*	F	α
PTL250	0.811469	0.209951	0.05	PTR250	0.375336	1.003157	0.05
PTL500	0.495685	0.713674	0.05	PTR500	0.155686	1.944767	0.05
PTL1000	0.429368	0.862691	0.05	PTR1000	0.093049	2.514093	0.05
PTL2000	0.256996	1.403613	0.05	PTR2000	0.201996	1.661997	0.05
PTL3000	0.383129	0.981636	0.05	PTR3000	0.078576	2.704156	0.05
PTL4000	0.53391	0.636999	0.05	PTR4000	0.023951†	4.083876†	0.05
PTL6000	0.165652	1.877073	0.05	PTR6000	0.013666†	4.763085†	0.05
PTL8000	0.023556†	4.10375†	0.05	PTR8000	0.026486†	3.963975†	0.05

Note: Statistical significance = alpha (α) 0.05.

PTL, pure tone left; PTR, pure tone right; F, F ratio.

† , statistically significant change.

The ANOVA results for the G-KCIN treatment in the left ear indicated statistically significant changes at 8000 Hz, whilst for the right ear, the results indicated statistically significant changes at 4000 Hz, 6000 Hz and 8000 Hz (*p* < 0.05), with changes progressively worsening as the frequencies increased. The significant changes occurred throughout the three sessions, with the mean pure-tone results increasing from session 1 to session 3. Therefore, hearing status changed at high frequencies for the patients on G-KCIN treatment, with both clinically and statistically significant changes at specified high frequencies.

### Ototoxicity monitoring in the bedaquiline treatment

**FIGURE 3:**
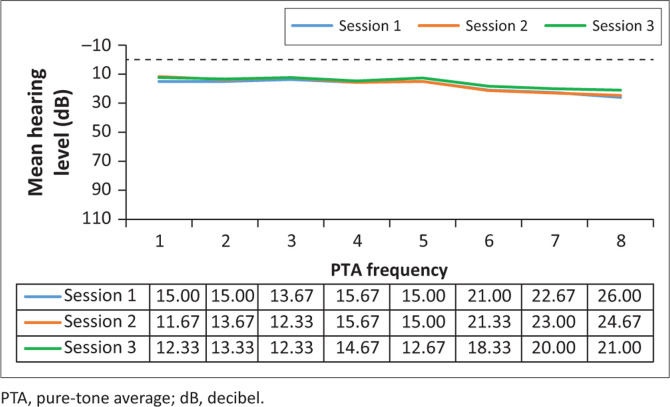
Mean left pure-tone audiometry results (in decibel) for the bedaquiline treatment, at the three different sessions (*n* = 15).

**FIGURE 4:**
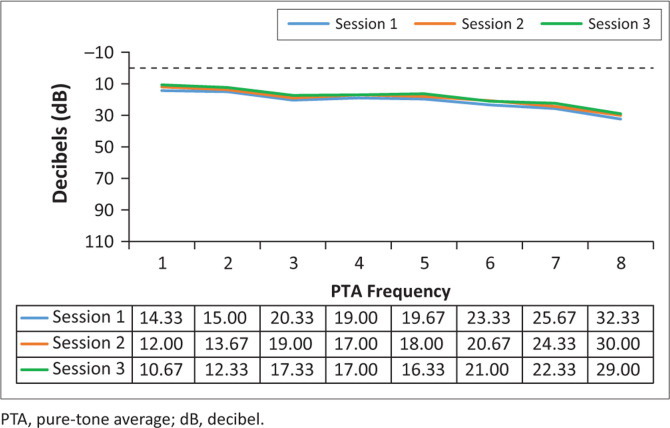
Mean right pure-tone audiometry results (in decibel) for the bedaquiline treatment, at the three different sessions (*n* = 15).

Analysis of the results for the G-BDQ (*n* = 15) involved comparing the pure-tone threshold results at all three sessions to determine whether any changes occurred during the period when they were receiving BDQ treatment. The pure-tone audiometry results in terms of means are depicted in Figures 3 and 4.

Clinically, mean hearing threshold results did not shift significantly between the sessions in this group, and this is indicative that the hearing status of the G-BDQ had not changed between the sessions, at all frequencies, bilaterally. Statistically, as depicted in Table 2, all the results demonstrated no statistically significant changes (*p*>0.05) across all frequencies tested.

**TABLE 2:** Results of analysis of variance for pure-tone audiometry for the bedaquiline treatment, indicating level of significance for ‘within group’ analysis.

Frequency	*p*†	F†	Α	Frequency	*p*†	F†	Α
PTL250	0.668846	0.406077	0.05	PTR250	0.342983	1.097808	0.05
PTL500	0.897226	0.108728	0.05	PTR500	0.391135	0.96	0.05
PTL1000	0.726586	0.321839	0.05	PTR1000	0.894843	0.111401	0.05
PTL2000	0.964229	0.036458	0.05	PTR2000	0.922119	0.081238	0.05
PTL3000	0.765116	0.269442	0.05	PTR3000	0.891102	0.115613	0.05
PTL4000	0.864055	0.146628	0.05	PTR4000	0.925872	0.077161	0.05
PTL6000	0.91795	0.114831	0.05	PTR6000	0.938994	0.063041	0.05
PTL8000	0.805211	0.217772	0.05	PTR8000	0.945184	0.056452	0.05

Note: Statistical significance = alpha (α) 0.05.

PTL, pure-tone left; PTR, pure-tone right; F, F ratio.

† , statistically significant change.

### Occurrence of tinnitus

Analysis of reports of tinnitus in the second and third follow-up sessions in both groups indicated that after the commencement of DR-TB treatment, there were more reports of tinnitus in the G-KCIN than in the G-BDQ. A total of six out of the 15 patients (40%) on G-KCIN treatment reported tinnitus unilaterally or bilaterally, whereas only one patient (7%) reported tinnitus in the group receiving bedaquiline.

### Comparison of the two groups

When establishing if there was a clear and measurable difference in audiological function in adults with DR-TB on bedaquiline over three assessment sessions when compared with those on G-KCIN, results from the two-sample, one-tailed *t*-test indicated that most of the cases rejected H_0_, implying that there was a statistically significant difference in hearing ability between both groups at all frequencies tested. A side-by-side comparison of the findings is depicted in Table 3. Clear clinically and statistically significant differences were found between the two groups, with adults on bedaquiline showing minimal to no changes in their hearing function over the treatment period.

**TABLE 3:** Table depicting comparison of results in both groups.

Variable	G-KCIN	G-BDQ
Ototoxicity monitoring (Pure- tone audiometry)	73.33% of the patients had a significant shift from baseline to their first follow-up in the left ear. 86.67% of patients had a significant shift from baseline to their second follow-up in the left ear. 80% of the patients had a significant shift from baseline to their first follow-up in the right ear. 93.33% of patients had a significant shift from baseline to their second follow-up in the right ear. Statistically significant changes at 8000 Hz in the left ear and at 4000 Hz, 6000 Hz and 8000 Hz in the right ear.	No shift, bilaterally. No statistically significant changes at all frequencies bilaterally.
Occurrence of tinnitus	Six of the 15 participants (40%) reported tinnitus.	One of the 15 participants (7%) reported tinnitus.

Note: A two-sample, one-tailed *t*-test indicated that most of the cases rejected H_0_, implying that there was a statistically significant difference in hearing ability between both groups at all frequencies tested.

G-KCIN, kanamycin treatment; G-BDQ, bedaquiline treatment.

## Discussion

Khoza-Shangase (2017) highlights important strategic indicators and variables that the audiology community needs to consider to play a more central role in preventive pharmaco-vigilance. The current study is located in this imperative as it seeks to evaluate the possible risk to hearing of a medication that has proven benefit for DR-TB management, an alternative to the documented ototoxic G-KCIN. Current findings, although from a small but age- and gender-matched sample size with a control group, reveal positive results on the part of hearing preservation during DR-TB treatment. In the current sample, the new DR-TB drug, bedaquiline, did not present to have ototoxic effects as indicated by the absence of clinically as well as statistically significant changes in the pure-tone audiometry results over three repeated longitudinal assessment sessions. Moreover, the fact that only one patient in the G-BDQ group was recorded to have complained of tinnitus also adds to these findings.

Current findings provide additional evidence on the known ototoxicity of G-KCIN, whilst providing new evidence on the possibly non-ototoxic nature of bedaquiline in the treatment of DR-TB. The pure-tone thresholds’ descriptive results indicating a decreased hearing function over time in patients on G-KCIN are not novel and are consistent with published evidence by Gamit (2014) on ototoxicity of G-KCIN in the treatment of DR-TB. However, the asymmetry of hearing loss presentation in the current sample does not have substantial evidential support in the literature, where one ear has been proven to be more susceptible to the effects of ototoxicity. Rachana and Shabnam (2017) do, however, state that even though ototoxicity typically results in bilateral, symmetrical hearing loss, there have been cases of unilateral and asymmetrical hearing losses reported. What these authors assert seems to be consistent with current findings, where the one ear presented with a slightly greater ototoxic reaction than the other ear.

These findings indicating worsening hearing thresholds (worsening ototoxicity) on G-KCIN at the second follow-up than at the post-baseline session are suggestive of the known cumulative effects of prolonged exposure to ototoxic medication. These findings are consistent with Khoza-Shangase’s (2020b) study, where hearing function of gold miners with and without a history of TB treatment presented similarly. They are also consistent with Modongo et al.’s (2014) findings where the degree of hearing loss and the risk of ototoxicity increased with increased duration of aminoglycoside treatment.

The finding indicating no ototoxic shift over time in patients receiving G-BDQ is a positive one within the South African context, where the prevalence of DR-TB remains high. The preservation of hearing function during DR-TB treatment with bedaquiline is agreeable with published evidence by Deoghare (2013), which indicated that ototoxicity was not one of the reported side effects of bedaquiline. Current findings, therefore, add to this limited evidence and can be used to motivate for more widespread prescription of bedaquiline, instead of G-KCIN, in this LMIC context. On benefit–risk evaluation, which is the process to ensure that all drugs are monitored in terms of their efficacy and analysed in terms of their negative side effects (Bankaitis & Schountz, 1998; Khoza-Shangase, 2017), the risk to hearing function seems to be significantly less with bedaquiline than with G-KCIN. These findings highlight the importance of carefully deliberating on benefit–risk evaluation of medications prescribed to treat diseases within LMICs. With recent evidence indicating that ototoxicity monitoring is gaining momentum within the South African research and clinical communities, although this is non-systematic, non-comprehensive and non-strategic in nature (Khoza-Shangase & Masondo, 2020), the value of benefit–risk assessments is highlighted by current findings.

The findings on the higher occurrence of tinnitus in the G-KCIN group compared with the bedaquiline group are consistent with published evidence by Mudd and Glatz (2018), indicating that tinnitus is a common side effect of ototoxic drugs such as G-KCIN. There is no published evidence on tinnitus in patients taking bedaquiline, so the current study findings are possibly the first indication of this otological symptom as a possible side effect of BDQ.

Although quality of life was not the focus of the current retrospective review, the findings of significant hearing loss with G-KCIN in this study seem to indicate that traditional DR-TB medication may decrease one’s quality of life, as hearing impairment and tinnitus have been linked to the decreased quality of life (Cone et al., 2018; Health Professions Council of South Africa, 2018; Ukaegbe, Orji, Ezeanolue, Akpeh, & Okorafor, 2017). The decreased quality of life is because of the hearing loss, making it difficult for the affected individual to understand the speech of others and engage in conversations, and consequently resulting in social isolation of the individual. As far as tinnitus is concerned, Ukaegbe et al. (2017) found that many individuals with tinnitus report anxiety, concentration difficulties, depression and irritability. The debilitating effects of hearing loss and tinnitus may even limit an individual from participating in certain activities of daily living, leading to social exclusion and isolation (Ukaegbe et al., 2017). As is well documented, hearing loss does have an effect not only on the affected individual but also on the individual’s communication partners, such as their family, friends and co-workers, known as ‘third-party disability’ (Packer, 2017). This is because hearing loss may lead to a communication breakdown, frustration, resentment and ultimately decreased interaction, thus often affecting relationships (Packer, 2017). Moreover, even though hearing aids can be used to improve one’s quality of life when ototoxicity has occurred, resource constraints within the South African public health sector, which are used by over 80% of the South African population, make this intervention not universally accessible to those needing it (Khoza-Shangase & Masondo, 2020). Current findings, therefore, on G-BDQ not being ototoxic in nature are positive and imply that the quality of life for the DR-TB-affected individuals and their families and communication partners will not be reduced as a result of debilitating hearing loss or tinnitus because of DR-TB treatment, as was the case with G-KCIN treatment.

## Conclusion

Current findings clearly demonstrated the positive benefits of the newly introduced DR-TB medication, bedaquiline, in as far as hearing preservation in a previously ototoxicity-ravaged population was concerned. Bedaquiline did not appear to cause an ototoxic shift as well as tinnitus in any of the patients who were taking it both clinically and statistically, and therefore, it seems to support the existing evidence, which states that the drug is not ototoxic. These findings support efforts to motivate for the widespread roll-out of this drug for DR-TB treatment in South Africa, as it is currently not universally accessible.

Current findings, although significant, should be interpreted bearing in mind the identified methodological limitations of the study. Firstly, the small sample size limits the generalisability; however, the fact that this was a longitudinal study with a control group raised the strength of the findings. Secondly, the retrospective design nature of the study prevented complete control over the data recorded. Methodological variables of the tester, such as the audiologist’s expertise, test technique and protocol, could have influenced the results. However, the fact that data were gathered from a specialised DR-TB treatment facility, where the clinical focus is on DR-TB, raises hopes that standardised protocols by experienced clinicians were adopted. Regardless of these limitations, current findings raise important implications for benefit–risk evaluations, resource allocation, policy formulation, graduate training and continued professional development of healthcare professionals involved in TB treatments. Finally, current findings raise implications for future studies.
